# Whole cell-based catalyst for enzymatic production of the osmolyte 2-*O*-α-glucosylglycerol

**DOI:** 10.1186/s12934-021-01569-4

**Published:** 2021-04-07

**Authors:** Katharina N. Schwaiger, Monika Cserjan-Puschmann, Gerald Striedner, Bernd Nidetzky

**Affiliations:** 1 Austrian Centre of Industrial Biotechnology (acib), Krenngasse 37, 8010 Graz, Austria; 2 Institute of Biotechnology and Biochemical Engineering, Graz University of Technology, NAWI Graz, Petersgasse 12, 8010 Graz, Austria; 3 Department of Biotechnology, University of Natural Resources and Life Sciences, Vienna, Muthgasse 18, 1190 Vienna, Austria

**Keywords:** 2-*O*-α-glucosylglycerol, Sucrose phosphorylase, Whole-cell biotransformation, High-yield protein expression, Fed-batch fermentation

## Abstract

**Background:**

Glucosylglycerol (2-*O*-α-d-glucosyl-*sn*-glycerol; GG) is a natural osmolyte from bacteria and plants. It has promising applications as cosmetic and food-and-feed ingredient. Due to its natural scarcity, GG must be prepared through dedicated synthesis, and an industrial bioprocess for GG production has been implemented. This process uses sucrose phosphorylase (SucP)-catalyzed glycosylation of glycerol from sucrose, applying the isolated enzyme in immobilized form. A whole cell-based enzyme formulation might constitute an advanced catalyst for GG production. Here, recombinant production in *Escherichia coli* BL21(DE3) was compared systematically for the SucPs from *Leuconostoc mesenteroides* (LmSucP) and *Bifidobacterium adolescentis* (BaSucP) with the purpose of whole cell catalyst development.

**Results:**

Expression from pQE30 and pET21 plasmids in *E. coli* BL21(DE3) gave recombinant protein at 40–50% share of total intracellular protein, with the monomeric LmSucP mostly soluble (≥ 80%) and the homodimeric BaSucP more prominently insoluble (~ 40%). The cell lysate specific activity of LmSucP was 2.8-fold (pET21; 70 ± 24 U/mg; *N* = 5) and 1.4-fold (pQE30; 54 ± 9 U/mg, *N* = 5) higher than that of BaSucP. Synthesis reactions revealed LmSucP was more regio-selective for glycerol glycosylation (~ 88%; position O2 compared to O1) than BaSucP (~ 66%), thus identifying LmSucP as the enzyme of choice for GG production. Fed-batch bioreactor cultivations at controlled low specific growth rate (µ = 0.05 h^−1^; 28 °C) for LmSucP production (pET21) yielded ~ 40 g cell dry mass (CDM)/L with an activity of 2.0 × 10^4^ U/g CDM, corresponding to 39 U/mg protein. The same production from the pQE30 plasmid gave a lower yield of 6.5 × 10^3^ U/g CDM, equivalent to 13 U/mg. A single freeze–thaw cycle exposed ~ 70% of the intracellular enzyme activity for GG production (~ 65 g/L, ~ 90% yield from sucrose), without releasing it from the cells during the reaction.

**Conclusions:**

Compared to BaSucP, LmSucP is preferred for regio-selective GG production. Expression from pET21 and pQE30 plasmids enables high-yield bioreactor production of the enzyme as a whole cell catalyst. The freeze–thaw treated cells represent a highly active, solid formulation of the LmSucP for practical synthesis.

**Supplementary Information:**

The online version contains supplementary material available at 10.1186/s12934-021-01569-4.

## Background

**Fig. 1:**
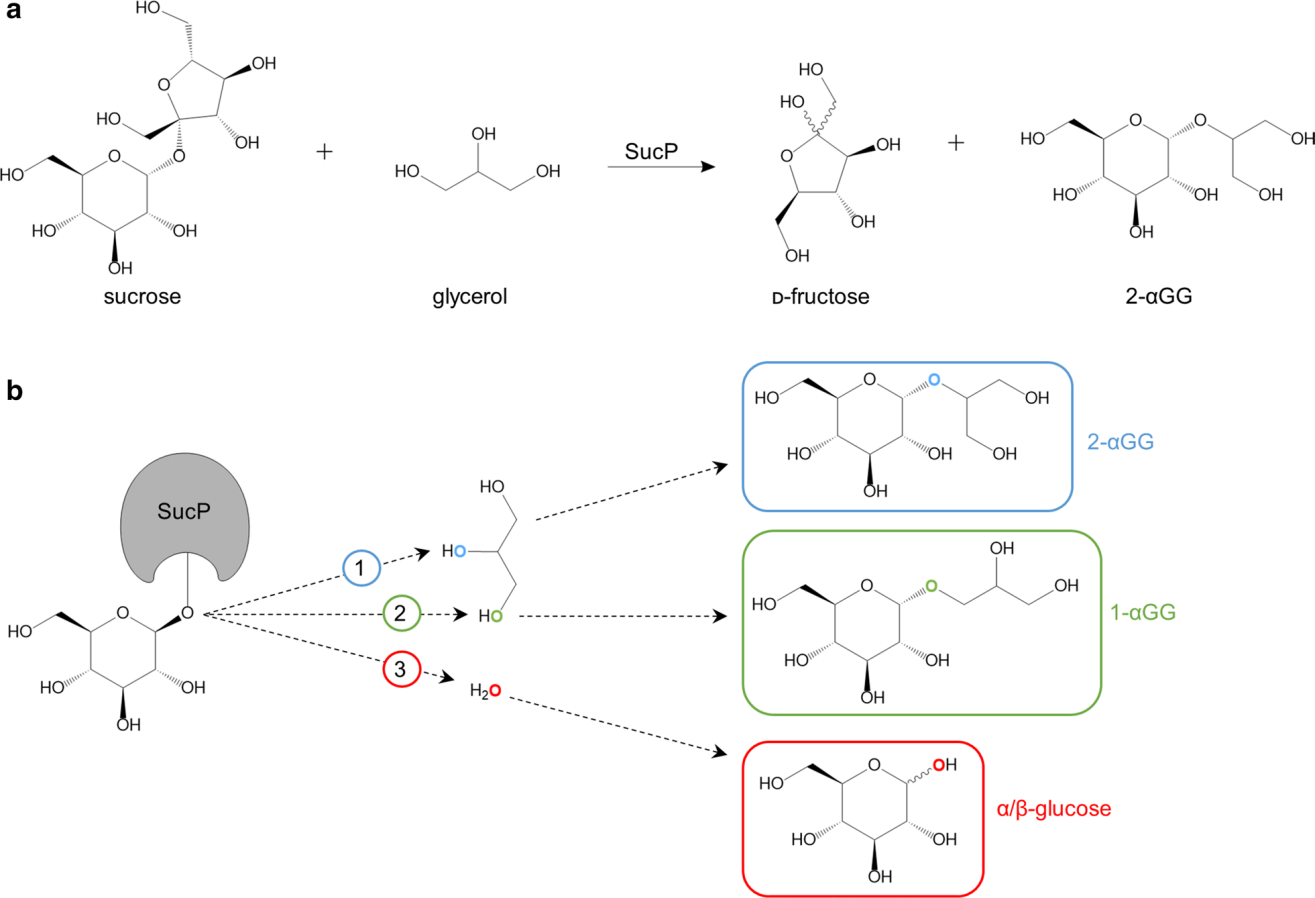
**a** Reaction for the enzymatic production of 2-αGG using sucrose phosphorylase (SucP). **b** Enzyme selectivity determines the product composition: (1) diastereoselectivity (2-αGG), (2) regio-selectivity (1-αGG), and (3) reaction selectivity (hydrolysis versus glycosylation). The atoms O1 and O2 of the glycerol molecule are marked in green and blue, respectively

The 2-*O*-α-d-glucopyranosyl-*sn*-glycerol (GG; Fig. 1a) is a natural compatible solute [1]. GG is prominently found in cyanobacteria that use it to counter the osmolality of saline environments [2, 3]. GG is additionally found in the resurrection plant *Myrothamnus flabellifolia* [4]. This desert plant can survive complete desiccation over years, without losing tissue integrity [5]. The protection of cells and tissues (e.g., skin) is an important general function of compatible solutes [6, 7], and this serves as the basis for promising applications in cosmetics [8, 9]. GG specifically offers high capacity of water binding, to generate a strongly moisturizing effect when applied on the skin [10]. Besides cosmetic applications, GG has significant interest for use as low-calorie sweetener [11] that can have a prebiotic effect [12].

To support development for industrial applications, GG must be made available efficiently at large scale. Microbial production of GG may be limited in the output parameters achievable from the biosynthesis. Specifically, product concentration (≤ 2 g/L), yield on substrates used (≤ 0.1 g/g), and productivity (≤ 0.04 g/L/h) are rather low in engineered *Corynebacterium glutamicum* [13] and even lower in engineered cyanobacterial *Synechocystis *sp. strains [14]. Process engineering may require specialized approaches for which “bacterial milking” can be an example [15, 16]. An efficient route of GG synthesis is glycosylation of glycerol from sucrose catalyzed by sucrose phosphorylase (SucP; Fig. 1a) [17]. Discovery of GG phosphorylases that catalyze 2-*O*-α-glucosylation of glycerol from β-d-glucose 1-phosphate [18, 19] or α-d-glucose 1-phosphate [20] has recently promoted alternatives of enzymatic GG synthesis, using two-enzyme phosphorylase cascade reactions. In these coupled reactions, either α-d-glucose 1-phosphate (αGlc1-*P*) or βGlc1-*P* is released in situ from sucrose or maltose to be used for glycosylation of glycerol [21]. Here, we are concerned with the simpler, single-enzyme reaction that yields GG directly from sucrose and glycerol (Fig. 1a).

The biocatalytic production relies on enzyme selectivity at three levels, as shown in Fig. 1b: diastereoselectivity for the α- or β-configuration in the product; regio-selectivity for position O2 or O1 in glycerol, and reaction selectivity (transfer to glycerol compared to hydrolysis). Used under suitable substrate conditions (e.g., 0.3–1.0 M sucrose; ≥ 1.5 M glyceol) as reported previously [22], the SucP from *Leuconostoc mesenteroides* (LmSucP) enables efficient GG production with useful three-tier selectivity [17, 23, 24]. Sucrose is an excellent donor substrate for glycerol glycosylation in high yield (≥ 90%) and with useful atom economy (~ 50%) [17]. GG is manufactured industrially by bitop AG (Dortmund, DE) using the biocatalytic process with LmSucP. GG is formulated into commercial product for cosmetic applications that is marketed as Glycoin^®^ (a 50% solution of GG).

The GG process underlines the importance of SucP as an industrial enzyme. Glycosylation by SucP offers considerable scope in the products made [25]. Facile formulation of SucP into a ready-to-use, potentially recyclable catalyst is thus of interest. SucP has been immobilized variously on solid support [26–31] and CLEAs (cross-linked enzyme aggregates) of the SucP from *Bifidobacterium adolescentis* have been prepared [32]. Whole cell-based catalyst of *Leuconostoc mesenteroides* was used to produce α-d-glucose 1-phosphate continuously from sucrose and phosphate [33]. Building on *Escherichia coli* as host for recombinant production of SucP, the analogous idea of whole cell biocatalysis was not explored. Considering the possibility of accumulating the SucP in large amounts in *E. coli*, the suitably formulated cells might constitute an attractive catalyst to be used in glycoside synthesis. Phosphorylase cascade reactions [21, 27, 34, 35] can likewise benefit from whole cell catalyst development.

Several studies show recombinant production of SucP enzymes in *E. coli* under standard conditions, apparently without major difficulties but also with quite variable efficiencies (see Table 1) [28, 36–43]. Systematic investigation of SucP expression has not been performed and inquiry focused on the development of an *E. coli*-based whole cell catalyst of SucP is lacking. We here therefore studied the expression of LmSucP and BaSucP in a comparative, side by side fashion. The two enzymes are the best characterized among the SucPs (for review, see [25]) and they furthermore represent different structural prototypes within this enzyme group: the LmSucP is a functional monomer [38] whereas the BaSucP folds into a tightly associated homodimer [44]. We speculated that difference in the oligomeric structure might affect the producibility of soluble enzyme in *E. coli*. We show that insoluble protein formation was by far less pronounced with LmSucP (≤ 20%) than with BaSucP (~ 40%). We also show that, compared to BaSucP, LmSucP was preferred for regio-selective GG production. Expression from pET21 and pQE30 plasmids gave a high-yielding bioreactor production of the enzyme as a whole cell catalyst. Freeze–thaw treated cells were highly active and readily usable for practical synthesis of GG.

**Table 1:** Sucrose phosphorylase expression data from literature and from this study

Enzyme	Native host	Expression strain	Specific activity of cell-free extract	% of total soluble protein	References
BaSP	*B. a.* DSM 20083	*B. adolescentis*	0.84 U/mg	0.80%	van den Broek et al. [36]
	*B. a.* DSM 20083	*E. coli*	19.356 U/L cell culture		van den Broek et al. [36]
	*B. a*. LMG 10502	*E. coli* Rosetta 2	1.5 U/mL cell culture (OD 1)	(~ 6%)	Aerts et al. [37]
	*B. a.* DSM 20083	*E. coli* BL21(DE3)	38 U/mg	33%	This study
LmSP	*L. m.* DSM 20193	*L. m.* DSM 20193	7 U/mg	3.7%	Koga et al. [38]
		*E. coli* DH10B	48 U/mg	25%	Goedl et al. [28]
		*E. coli* BL21(DE3)	55 U/mg	30%	This study
	*L. m.* ATCC 12291	*E. coli* 1101 (slp-*spl*-1)	55.7 U/mg	30%	Kitoa et al. [39]
	*L. m.* No. 165	*E. coli* JM109	4.6 U/mg	50%	Kawasaki et al. [40]
	*L.m*	*E. coli* BL21(DE3)	~ 22 U/mg CDM (~ 44 U/mg CFE)	25%	Su et al. [41]
	*L. m.* NRRL B-1149	*E*. *coli* BL21(DE3)pLysS	1.49 U/mg	Specific activities of enzymes	Lee et al. [43]
	*L. m.* NRRL B-742	*E. coli* BL21(DE3)pLysS	1.8 U/mg		Lee et al. [42]
TtSP	*Thermoanaerobacterium thermosaccharolyticum*	*E. coli* BL21(DE3)	25.7 U/mg	50%	Yao et al. [85]
BlSP	*Bifidobacterium longum*	*E. coli* BL21	13 U/mg	11%	Hui Zhang [86]
		*E. coli* JM109	12.5 U/mg	10%	Shin et al. [87]

## Results and discussion

### Recombinant production of LmSucP and BaSucP with different expression systems

The LmSucP gene (GenBank identifier: D90314) is 1788 bp in size and encodes a protein of 55.75 kDa. The BaSucP gene (GenBank identifier: AF543301) is 2400 bp in size and encodes a protein of 56.20 kDa. Each protein is appended either at its authentic N-terminus (pQE30) or its authentic C-terminus (pET21) with a tag of 6 histidines. The specific activities of the N- and C-terminally tagged enzymes are 118 ± 6 U/mg (mean of *N* = 6) and 120 ± 8 U/mg (mean of *N* = 8) for BaSucP, respectively. They are 177 ± 11 U/mg (*N* = 7) and 174 ± 10 U/mg (*N* = 5) for LmSucP, respectively. Literature values (120 U/mg for C-terminally tagged BaSucP [45]; 190 U/mg for N-terminally tagged LmSucP [28]) were confirmed from this study.

The plasmid vectors pQE30 and pET21 were used for expression (Additional file 1: Figure S1). Both vectors are well characterized for recombinant protein production [46]. The main differences between the two are the following: The T7_*lacO*_ promoter of pET21 is considered stronger than the T5_*lacO*_ promoter in pQE30 [47–49]. Genes encoding *LacI* (*lac* operon repressor) and Rop (repressor of primer) are present in pET21 but lacking in pQE30. These structural changes in plasmid imply a less tightly controlled expression from pQE30 than pET21 caused by the absence of *LacI*; and a 2 to 3-fold higher copy number of pQE30 compared to pET21 (15–50 copies per cell) [50–52] caused by the absence of Rop [53, 54], despite usage of the same origin of replication (pBR322). *E. coli* BL21(DE3) was used as the expression host. Induction was done identically with both vector systems, using isopropyl-β-d-thiogalactoside (0.25 mM) and at 25 °C. In shake flask cultivations, the maximum specific growth rate of *E. coli* BL21(DE3) during induction was measured (Additional file 1: Figure S2) and found to vary little with combination of insert and plasmid used (~ 0.72 h^−1^), except for BaSucP_pET21 that caused slower growth (0.55 h^−1^).

**Fig. 2:**
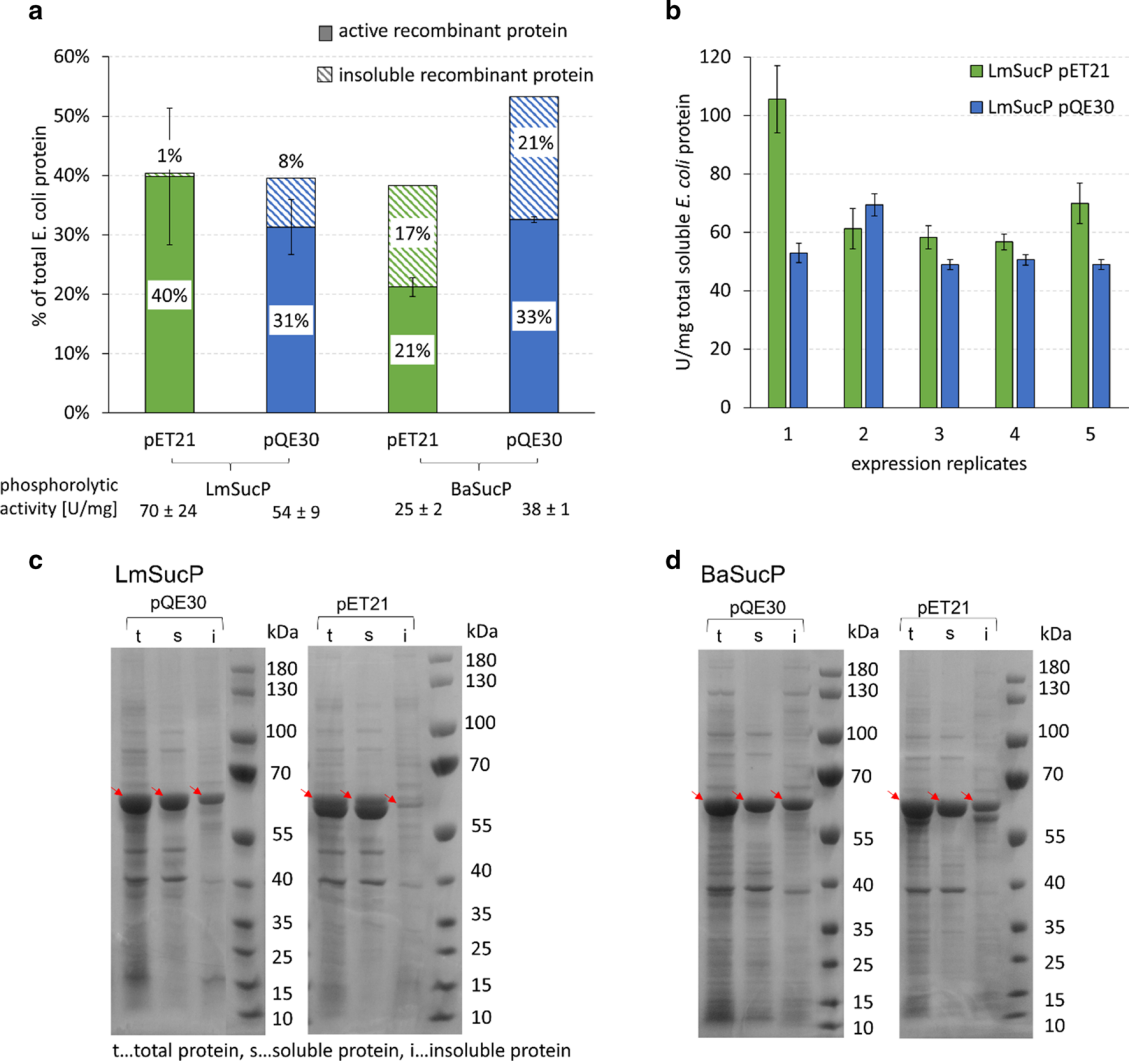
Results of expression analysis. **a** Soluble and insoluble recombinant protein (LmSucP and BaSucP) percentage share of total soluble *E. coli* protein expressed by pQE30 and pET21 and corresponding phosphorolytic activity (U/mg total *E. coli* protein). One unit (U) of activity is the enzyme amount producing 1 µmol αGlc1-*P*/min under the conditions used. Standard deviation (±) was calculated from at least three expression replicates. **b** Expression replicates of LmSucP from pET21 and pQE30, standard deviation was calculated from at least three analytical determinations. SDS PAGE gels of one representative expression replicate for **c** LmSucP (56.81 kDa) and **d** BaSucP (57.50 kDa), respectively (see also Additional file 1: Figure S4)

Each insert-vector combination was characterized in two ways. Using SDS PAGE with densitometric analysis of protein bands (Additional file 1: Figure S3), the portion of recombinant enzyme in the insoluble protein fraction was quantitated relative to the soluble fraction from lysed cells (Fig. 2a). Secondly, the specific enzyme activity in the cell lysate was measured (Fig. 2a, b). LmSucP accumulated ~ 40% of total protein and nearly all (pET21) or most (≥ 80%; pQE30) of it was soluble (Fig. 2a, c). Production of BaSucP proceeded to higher levels using pQE30 (~ 54% of total protein) compared to pET21 (~ 38% of total protein) (Fig. 2a). Insoluble protein formation was more pronounced for BaSucP (pET21: ~ 45%; pQE30: ~ 39%) than it was for LmSucP (Fig. 2a, d). Specific activities obtained from three to five serial expression replicates were well consistent for BaSucP, giving 25 ± 2 U/mg (*N* = 3) and 38 ± 1 U/mg (*N* = 3) for the expression from pET21 and pQE30, respectively. For reasons unknowable from the current study, the specific activities of LmSucP showed larger variation (± ~ 20%) across the serial replicates (Fig. 2a, b) than those of BaSucP. Using pET21 in particular (Fig. 2b), the specific activity of LmSucP started from a value of more than 100 U/mg in the first expression, which decreased to its almost half (60 U/mg) in the subsequent expression (Fig. 2b). On average, the specific activities were 62 ± 5 U/mg (*N* = 4; which includes only rounds 2–5 in Fig. 2b) and 54 ± 9 U/mg (*N* = 5) for the expression from pET21 and pQE30, respectively.

Table 1 summarizes the results of this study along with selected literature data on the production of LmSucP and BaSucP in the native organism or in *E. coli*. Differences in the conditions used make it impossible to compare the individual studies directly. Table 1 is nonetheless important to show the range of specific and volumetric activities obtained from the different approaches. Regarding enzyme production for high titer, the pQE30-based expression seems promising for both LmSucP and BaSucP. pQE30 is preferred over pET21 because expression was stronger with BaSucP and less variable in the specific activity obtained from LmSucP.

**Fig. 3:**
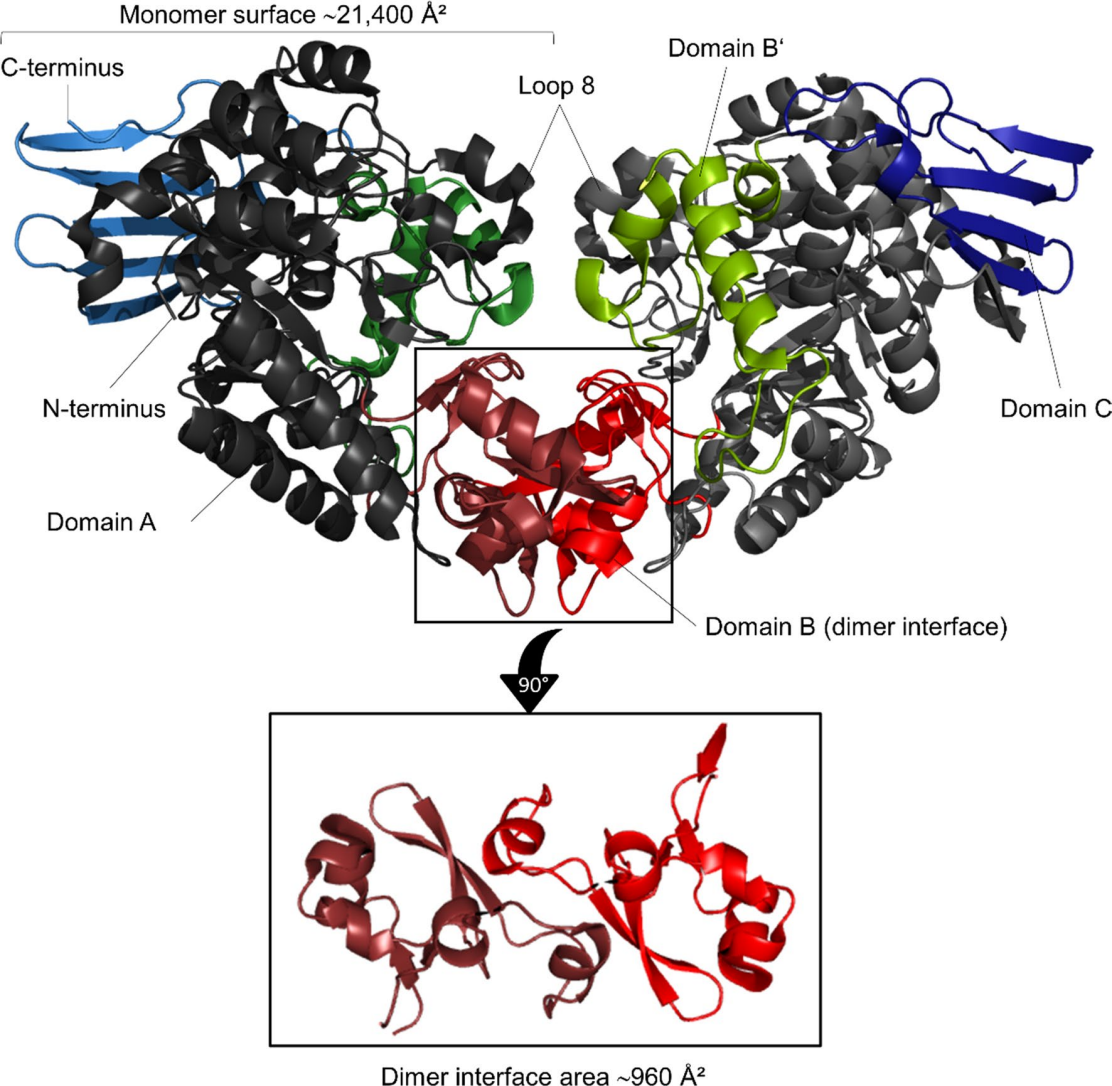
Crystal structure of the homodimeric BaSucP (PDB entry 1r7a). Domain A is displayed in grey, domain B in red, domain Bʹ in green and domain C in blue. Most dimer interactions are confined to the two B domains (bottom), but interactions between loop 8 regions of the two domain A barrels have also been observed previously [44]

We considered reasons why in the case of BaSucP the expression from pQE30 gave better results than the expression from pET21. Interestingly, it has been reported that the location of the His-tag (N- or C-terminus) affects the expression levels of recombinant proteins [55–57]. A more recent study even observed a distinct over-expression of proteins with N-terminal His-tags before induction (over 25% of total cellular protein) [58]. Hence, the N-terminal His-tag, together with the additional phase of low-level non-induced expression, that both only the expression from pQE30 involved, might be responsible for the higher BaSucP production. Moreover, in the phase of non-induced expression, as shown in Additional file 1: Figure S4B, most of the BaSucP was completely soluble. Therefore, the time for proper folding must have been sufficient for the recombinant enzyme formed and the concentration of aggregation-prone folding intermediate cannot have exceeded the solubility limit. The relatively smaller degree of insoluble protein received with LmSucP compared to BaSucP can arguably be ascribed to the more complex folding requirements of the latter enzyme, which is a functional homodimer. Sprogøe et al. [44] showed with the crystal structure of BaSucP that ~ 4% (= ~ 960 Å^2^) of the total monomer surface area (~ 21,400 Å^2^) was buried within the dimer interface (see also Fig. 3). According to Nussinov [59], monomeric folding intermediates are stable when they are compactly folded and the monomer surface area buried upon dimerization is small. Protein compactness can be expressed from the ratio of monomer surface area and amino acid chain length. For BaSucP, compared to some other proteins [59], the compactness parameter (41 = 21,400/519) together with the 4% buried surface area indicate that folding of the enzyme is likely to pass through a rather stable, monomeric intermediate [59, 60]. However, under conditions of induced expression, such intermediates can easily accumulate to levels that promote protein aggregation into an insoluble precipitate. Lastly, Additional file 1: Figure S4B shows evidence of protein degradation in the BaSucP produced by expression from pET21. A double band was visible at the BaSucP position in the gel when the whole *E. coli* protein or just the insoluble protein was analyzed. The protein band slightly smaller than the native BaSucP has likely arisen from partial proteolysis of the full-length protein at the intermediate stage of folding. Note that proteolytic degradation rarely happens in the insoluble protein [61].

### Regioselective glycosylation of glycerol for efficient synthesis of GG

**Fig. 4:**
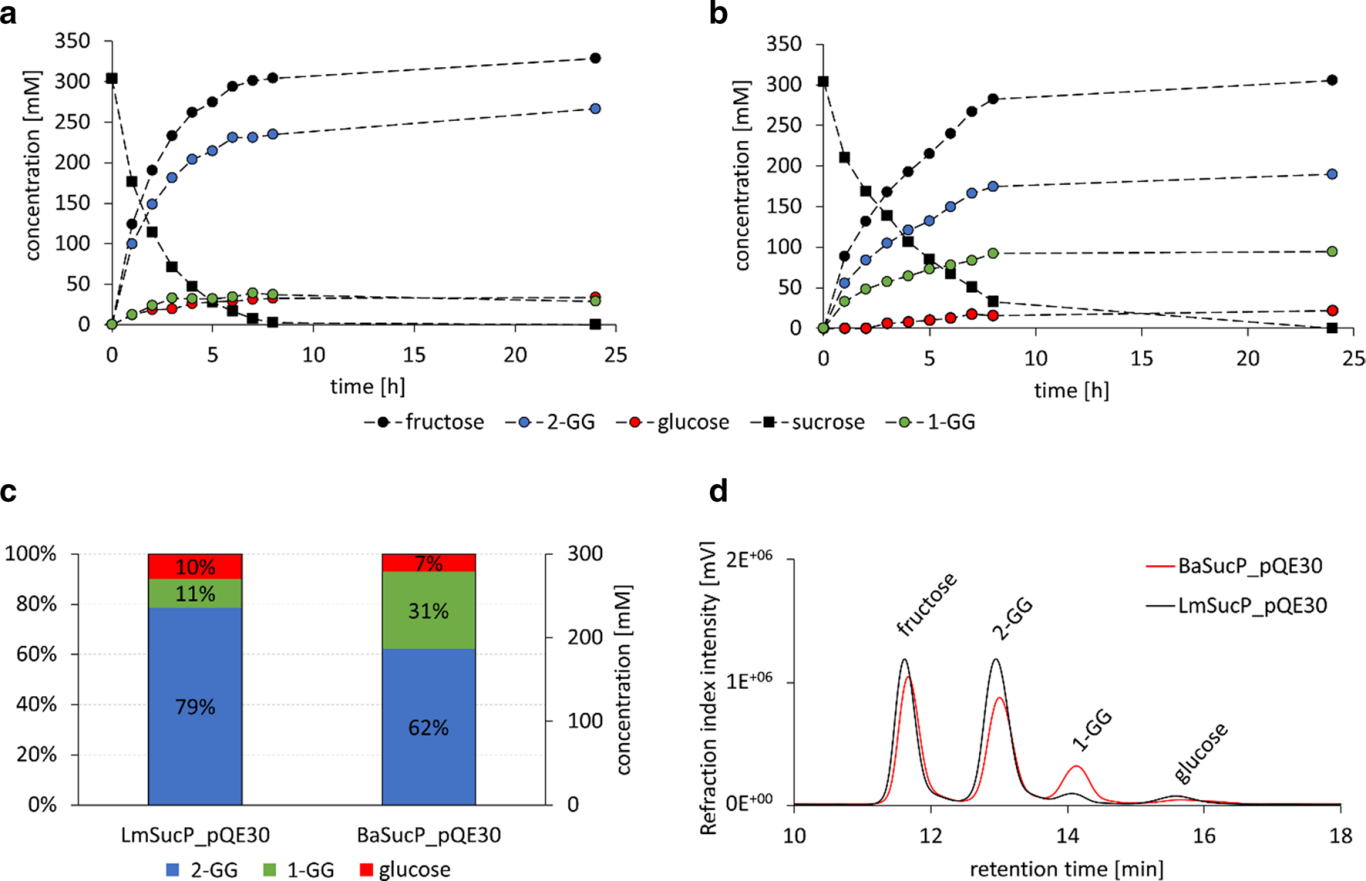
Bioconversions of **a** LmSucP_pQE30 and **b** BaSucP_pQE30 cell-free extracts. Reaction conditions: 300 mM sucrose, 1800 mM glycerol, 40 U/mL sucrose phosphorylase (phosphorolysis direction of the respective cell-free extract), 50 mM MES buffer at pH 7, 30 °C, 300 rpm for 24 h. **c** Product composition after 24 h GG production and **d** corresponding chromatograms of HPLC measurements

High-level expression for enzyme production in *E. coli* is useful in general to promote the various applications of LmSucP and BaSucP in glycoside synthesis [21, 25, 34, 62]. To select the most promising candidate for the whole cell-catalyzed GG production, we first performed synthetic reactions using the cell lysates. Results of a comprehensive time course study are summarized in Fig. 4. A dedicated HPLC method was used to separate the relevant compounds for quantification in a single-run analysis (Fig. 4d). GG is distinguished from its regioisomer 1-*O*-α-d-glucosyl glycerol (1-GG). Glycerol consumption was measured but is not shown, as it is related to the appearance of GG and 1-GG by mass balance. The results show the following (Fig. 4 and Additional file 1: Table S1): (1) Sucrose was used completely in both reactions. In the case that complete (≥ 95%) conversion was demanded, the LmSucP reaction was faster (~ 1.3-fold). (2) Both reactions released glucose due to hydrolysis of sucrose. Especially in the early reaction phase (~ 5 h), LmSucP produced more glucose than BaSucP. This observation was consistent with a recent kinetic study that showed BaSucP to exhibit higher reaction selectivity (glycosylation of glycerol compared with hydrolysis) than LmSucP [63]. (3) Both enzymes formed 1-GG as secondary glycosylation product to GG. The portion of 1-GG in the product was 11% or smaller for LmSucP but reached 31% for BaSucP (Fig. 4c). The regioselectivity of neither enzyme was perfect but that of BaSucP was not sufficient for GG production.

**Fig. 5:**
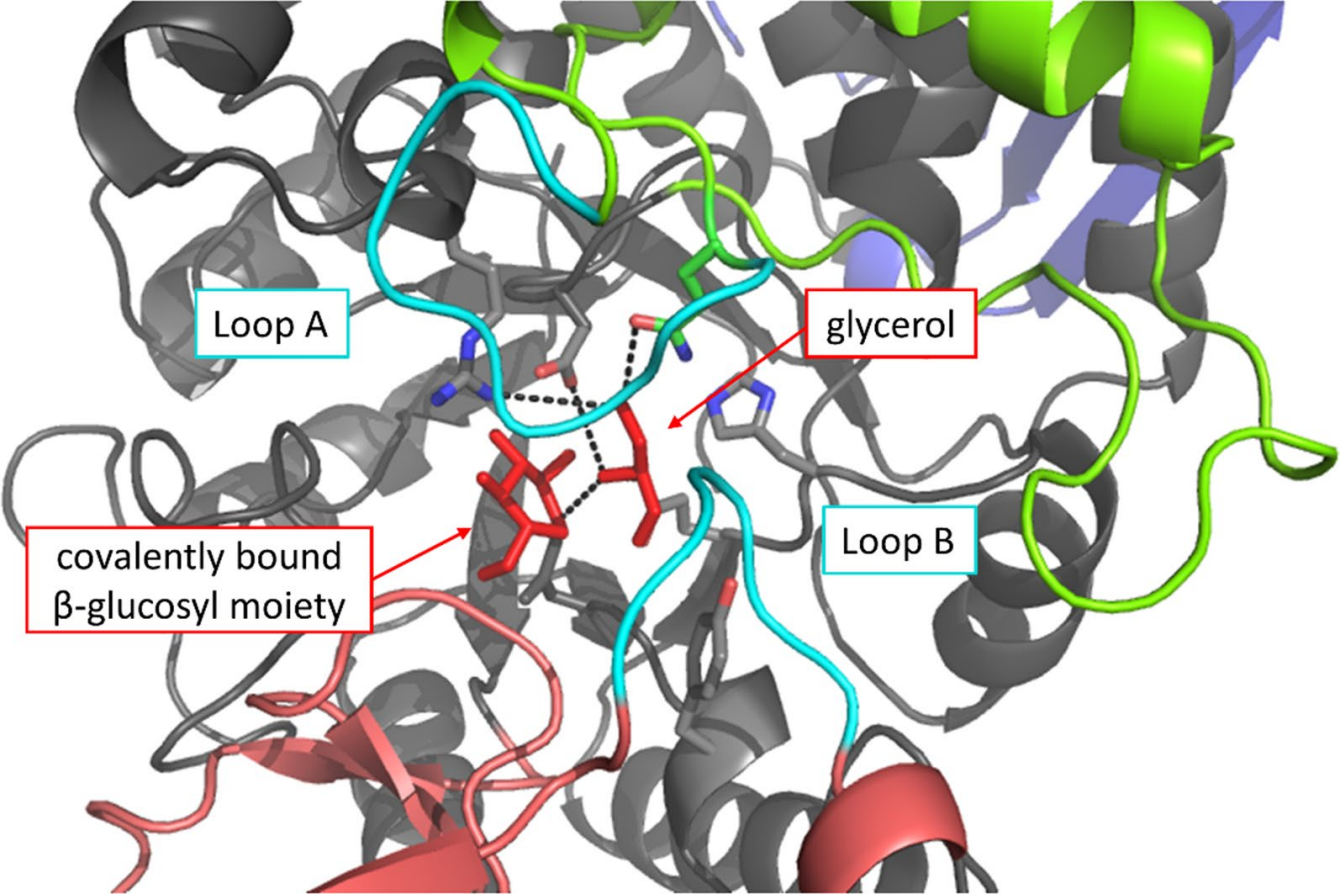
Positioning of glycerol in BaSucP for regioselective glycosylation at O2. Illustration of mobile loops (cyan) and binding pocket for acceptor substrate of the β-glucosyl enzyme intermediate of BaSucP (PDB-entry 2gdv, molecule A). Interactions of a previously performed glycerol acceptor docking experiment [67] are shown. Color code for enzyme domains corresponds to Fig. 3

Glycerol docking into the structure of the covalent β-glucosyl enzyme intermediate of LmSucP (modelled from the experimental BaSucP structure) has previously suggested a plausible mode of glycerol binding for glycosylation at its O2 [64]. The alternative mode of glycerol binding for glycosylation at its O1 is unknown. The BaSucP structure reveals two mobile loops that line the binding pocket for the acceptor substrate (Fig. 5). These loops undergo structural rearrangement during the catalytic cycle to enable binding of both fructose and phosphate in the canonical reaction of the enzyme (phosphorolysis of sucrose) [45, 65, 66]. It stands to reason based on chemical intuition that the glycerol acceptor would be accommodated rather in the acceptor binding pocket shaped for fructose binding than in the pocket shaped for phosphate binding. However, the high structural flexibility of the acceptor binding pocket might allow for glycerol binding in different orientations and could thus be responsible for the low regioselectivity of BaSucP in the reaction with glycerol. The amino acids making the acceptor binding pocket of the BaSucP structure are well conserved in LmSucP [24]. Structural interpretation of enzyme regioselectivity different in LmSucP and BaSucP must await evidence on glycerol binding at atomic resolution in at least one, but ideally both enzymes.

### Controlled bioreactor cultivation for LmSucP production

**Fig. 6:**
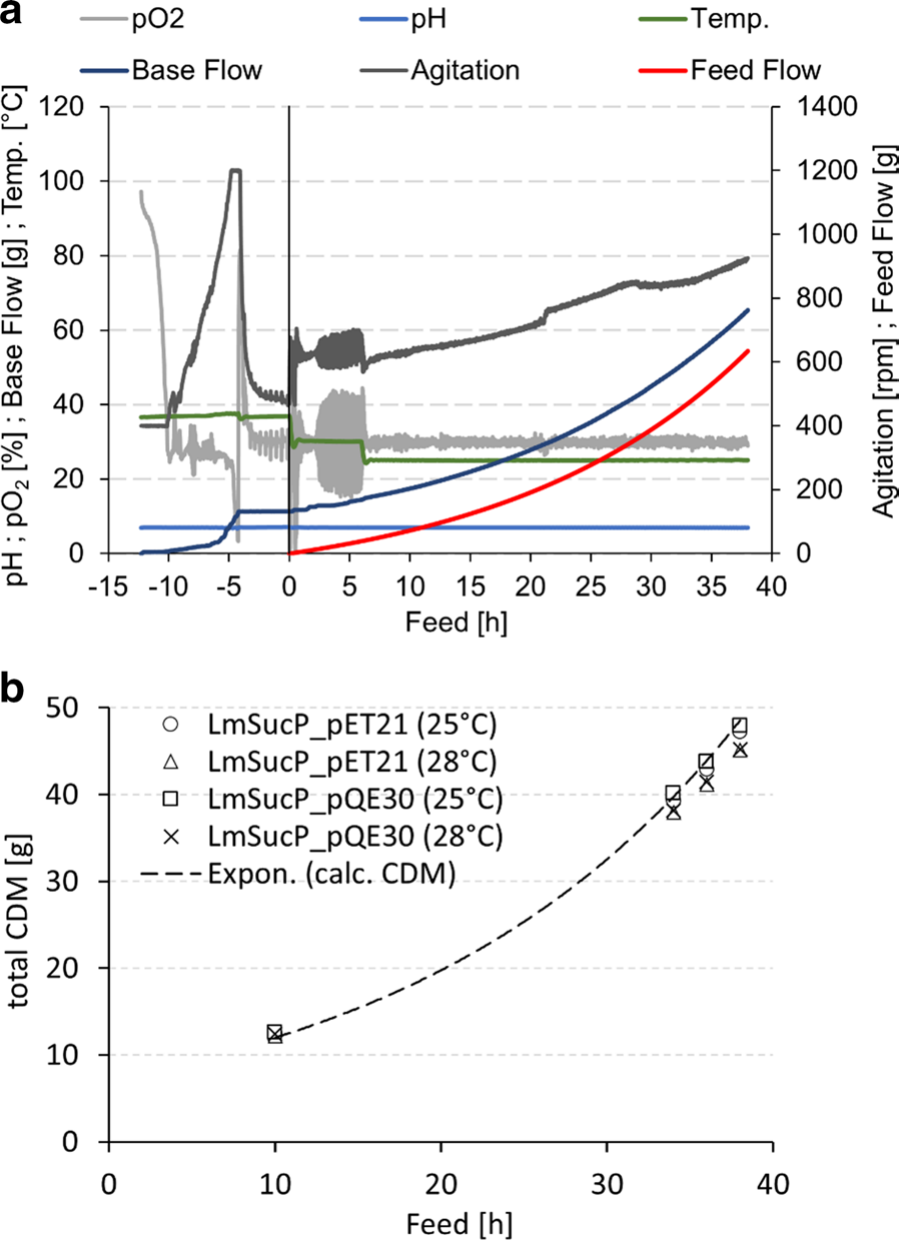
Fed-batch bioreactor cultivation. **a** Example time course with control parameters shown (LmSucP_pET21 at 25 °C). **b** Cell dry mass formation and calculated cell dry mass for the different cultivations

To increase the cell concentration in the *E. coli* cultivation, we performed carbon-limited fed-batch bioreactor experiments (1.5 L; working volume 1.2 L) using minimal medium. By using an exponential substrate feed, the specific growth rate µ was adjusted to 0.05 h^−1^. This µ was 6 times smaller than the average µ (0.3 h^−1^) in the shake flask cultivation. Strains harboring LmSucP_pQE30 and LmSucP_pET21 were assessed in parallel experiments at 25 °C and 28 °C. The conditions were chosen because of results from shaken flask experiments suggesting a critical influence of cultivation temperature in just that range, as discussed below. An exemplary description of the measurement and control parameters in the course of time of all four bioreactor cultivations is given in Fig. 6a. The cells could maintain the predefined growth rate over almost the whole production phase, only a marginal deviation was observed at the end of the cultivation, as shown in Fig. 6b. In all cultivations, irrespective of the plasmid vector and the temperature used, an *E. coli* cell density of ~ 40 g/L, equivalent to ~ 50 g CDM, was reached. The used cultivation and induction strategy led to soluble LmSucP production and gave high volumetric yields (up to 4 g/L) at the end of the process. Excessive formation of insoluble protein was not observed.

**Fig. 7:**
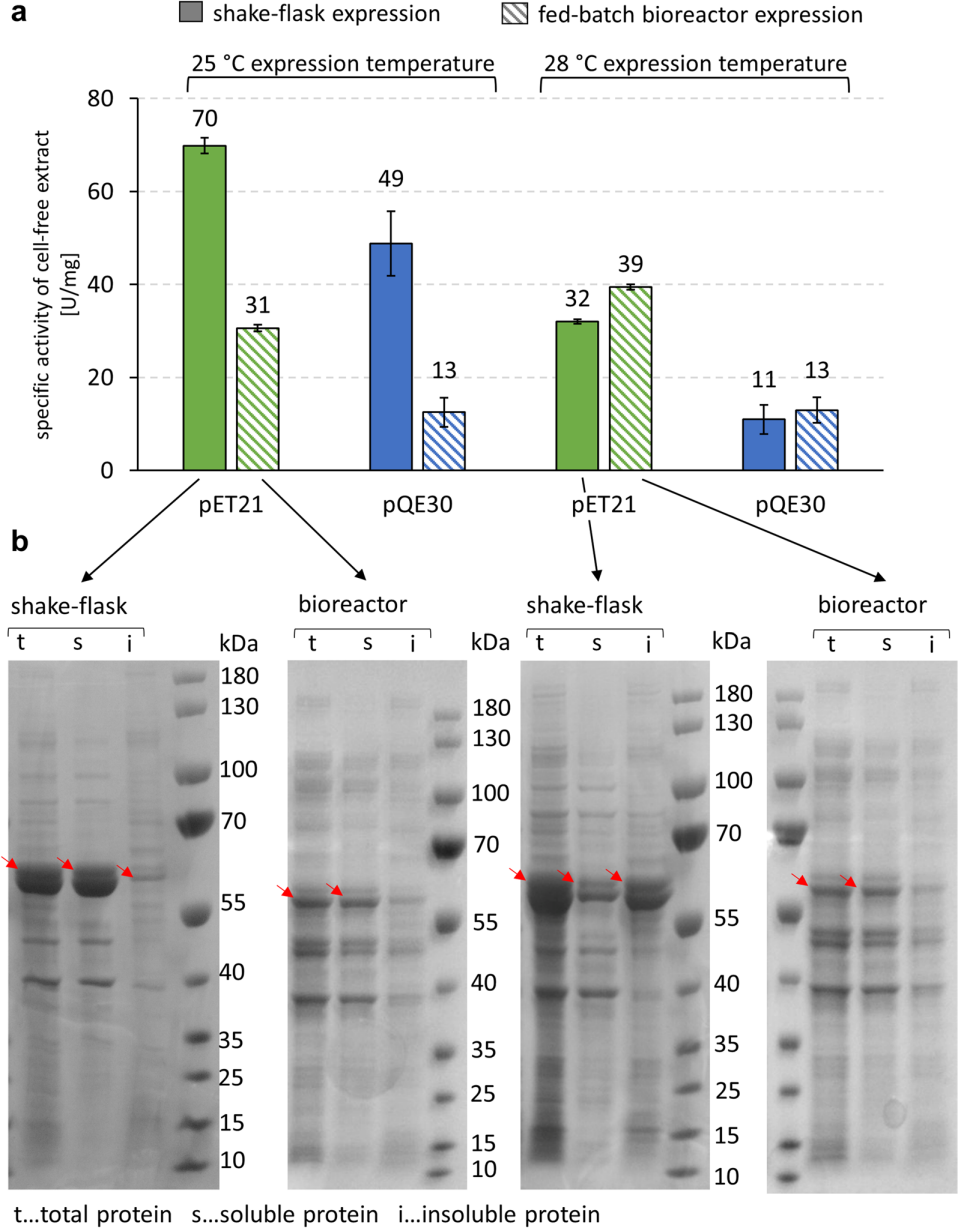
Fed-batch bioreactor expression versus shake-flask expression of LmSucP_pQE30 and LmSucP_pET21 at two expression temperatures (25 °C and 28 °C). **a** Specific phosphorolytic activities of cell-free extracts. Standard deviations were calculated from at least three analytical determinations. **b** Corresponding SDS PAGE gels of LmSucP_pET21

Enzyme production in shaken flasks at 28 °C gave a considerably lower specific activity of LmSucP (pET21: 2.2-fold; pQE30: 4.4-fold) compared to enzyme production at 25 °C (Fig. 7a). The effect can be ascribed to a substantially larger portion of LmSucP found as insoluble protein at 28 °C than at 25 °C (Fig. 7b). Remarkably, an increase in temperature by just 3 °C caused the soluble/insoluble protein ratio for LmSucP to become inverted at 28 °C (Fig. 7b, Additional file 1: Figure S5). The lack of measurement and control in the shake flask cultivation makes it difficult to identify the possible origin of what we consider an unusual temperature sensitivity of the recombinant protein production in *E. coli*. Interestingly, in terms of LmSucP specific activity achieved, enzyme production in the bioreactor was largely independent of the temperature in the range studied (Fig. 7). Presumably, in consequence of the restricted growth rate due to feed control, the formation of insoluble recombinant protein was completely suppressed at both 25 °C and 28 °C in the bioreactor (Additional file 1: Figure S6). The specific activities from the bioreactor cultivations were lower than from shake flask cultivations at 25 °C (pET21: 2.2-fold; pQE30: 3.8-fold) but comparable at 28 °C (Fig. 7a). Overall, normalized on the working volume (1.2 L), the fed-batch bioreactor production yielded 7.6 × 10^5^ U/L and 2.5 × 10^5^ U/L LmSucP using expression from pET21 and pQE30, respectively. The corresponding productivity was 3.2 × 10^4^ U/h and 1.0 × 10^4^ U/h (28 h after induction) and the whole cell activity normalized on the cell dry mass was 2.0 × 10^4^ U/g and 6.5 × 10^3^ U/g. Even at this stage of development without dedicated optimization, the enzyme production in the fed-batch bioreactor can be considered efficient. The specific enzyme activity in cell lysate was retained largely while taking the LmSucP production from the shake flask to the bioreactor. However, high sensitivity of the soluble/insoluble protein ratio in the shake flask production to tiny change in temperature (25 °C → 28 °C) was an unexpected phenomenon. Since the same temperature effect was completely absent in the bioreactor cultivation, it could not have originated from the enzyme intrinsically. The fundamental problem of unknown system parameter(s) affecting the output of recombinant protein production in the shake flasks is therefore emphasized. These results strongly support early integration of bioreactor studies into the development of recombinant enzyme production in *E. coli*.

### Whole cell-based catalyst for GG production

Assuming an overall protein concentration in the *E. coli* cytoplasm of ~ 300 g/L [68], we can use the result from Fig. 2a (~ 40% LmSP of total intracellular protein) to estimate an enzyme concentration of ~ 120 g/L in induced *E. coli* BL21(DE3) cells harboring LmSucP_pET21 or LmSucP_pQE30. Considerable processing effort would be necessary to establish a similarly concentrated state for the (partially) isolated enzyme. However, whole cell-based enzyme formulations can retain the LmSucP highly concentrated as produced by recombinant protein biosynthesis. Moreover, the number of processing steps for catalyst preparation can be reduced when whole cells are used. Permeabilization of the cells is usually necessary to make available the intracellularly localized enzyme activity for the synthetic reaction, unmasked from effects of transport across the cell membrane. A large set of methods exists for cell permeabilization [69–74]. A balance between unmasking the enzyme activity and enzyme leakage from the cells must be achieved. We here focused on “physical permeabilization” by freezing and thawing or freeze-drying. Both processes are mild in destabilizing the whole cell integrity [75, 76]. They are benign in that organic solvents are not used. Chemicals (e.g., detergents) that are difficult to remove later are not introduced.

**Fig. 8:**
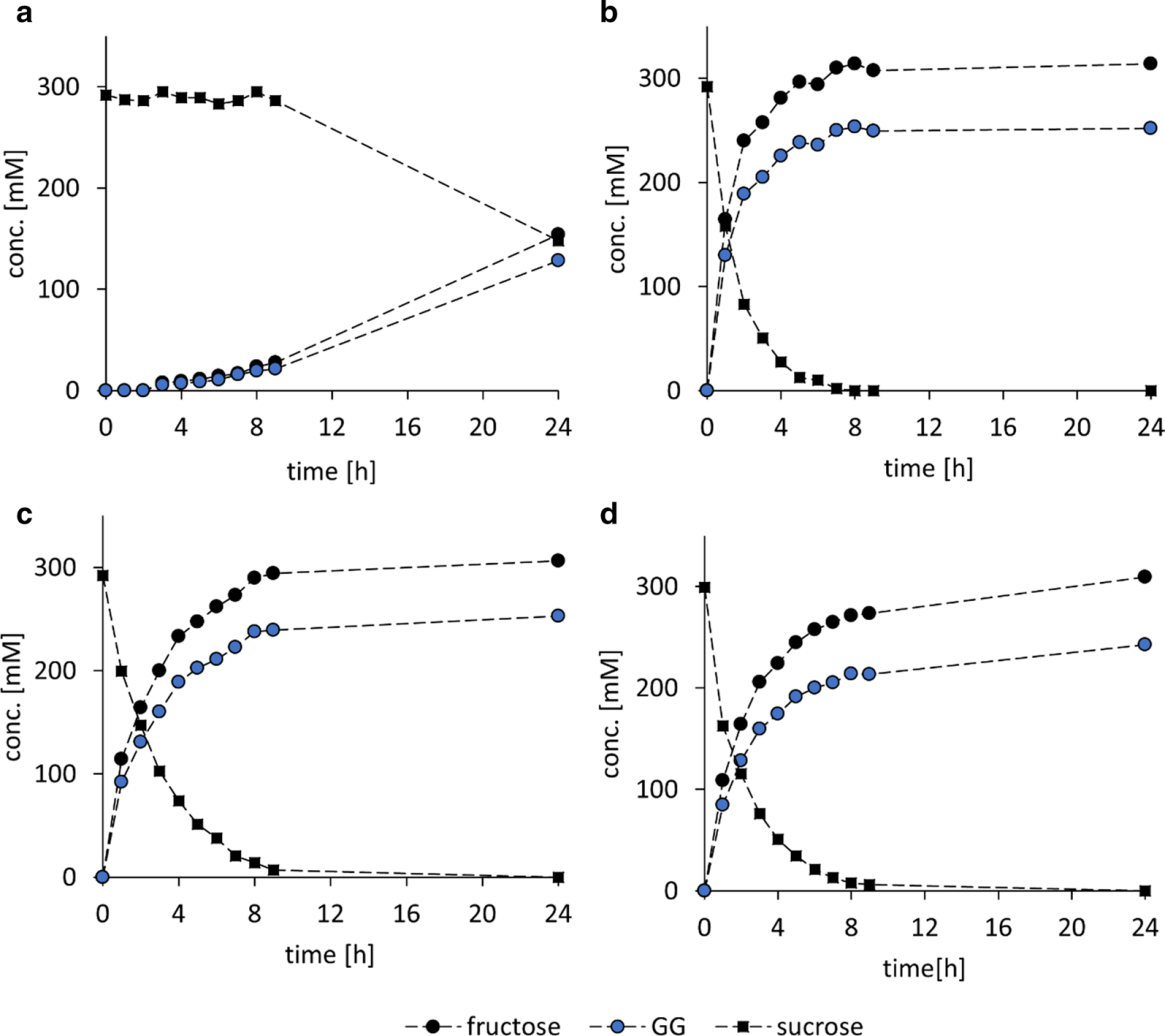
Evaluation of different catalyst preparations of *E. coli* LmSucP_pQE30 in GG synthesis. **a** Non-permeabilized cells, **b** cell lysate, **c** freeze-thawed cells, and **d** freeze-dried cells. Reactions were performed using 300 mM sucrose, 1.8 M glycerol, 40 U/mL LmSP phosphorolysis activity, 50 mM MES buffer at pH 7, 30 °C, 300 rpm for 24 h

To assess the different whole cell preparations of LmSucP for GG production, we performed synthetic reactions and evaluated the performance against cell lysate as the reference. Reaction time courses are shown in Fig. 8 and key parameters of conversion efficiency calculated from the data are summarized in Table 2. The non-permeabilized cells expressed only ~ 14% of the reference activity (850 U/g CDW). Freeze-thawing and freeze-drying were similar in achieving ~ 71% and ~ 65% of the reference activity. However, enzyme leakage to supernatant was lower for freeze-thawed cells (~ 1%) compared to freeze-dried cells (~ 5%). The freeze-thawed cells may thus be suitable for encapsulation to facilitate recycling. The space–time yield after ~ 95% sucrose conversion was slightly higher (1.6-fold) with the cell lysate compared to freeze-thawed whole cells (Table 2). The final GG yield was however similar with both catalyst preparations. In summary, freeze–thaw treatment appears to be suitable for *E. coli* cell permeabilization. It yields a LmSucP whole cell catalyst that is highly active and avoids enzyme leakage from the solid material.

**Table 2:** Efficiency parameters of different cell catalysts (LmSP_pQE30) preparations in GG synthesis

Treatment	GG yield		Catalyst activity^a^	GG concentration	Space–time yield^b^	Reaction^c^/site^d^ selectivity
	2 h (%)	24 h (%)	U/g_CDM_	g/L	g/L/h	%
Non-permeabilized	0	44	11.5	32.6	1.4	93/88
Extracted	65	86	846	64.0	12.1	89/88
Freeze-thawed	45	86	598	64.3	7.6	92/88
Freeze-dried	42	80	553	61.7	7.5	87/88

^a^One U is the enzyme amount producing 1 µmol GG/min

^b^Calculated for a sucrose conversion of 95%, except for non-permeabilized cells, where conversion was 50%

^c,d^Percent ratio of ^c^ GG formed/Fru released (= sucrose converted) and ^d^ GG/(1-GG + GG)

## Conclusions

This systematic study of plasmid-based expression in *E. coli* of LmSucP and BaSucP shows accumulation of recombinant protein to levels of ~ 50% of total intracellular protein. The simpler monomeric structure of LmSucP compared to the homodimeric structure of BaSucP favors soluble expression. Specific phosphorolytic activities of up to 35,000 U/g CDM (corresponding to 70 U/mg total protein) were obtained, supporting the development of a whole cell-based catalyst of both enzymes. For GG production, LmSucP is the preferred choice due to its higher regioselectivity compared to BaSucP. The enzyme production for LmSucP was scalable to fed-batch bioreactor cultivation (1.2 L volume) that resulted in ~ 40 g CDW/L and 7.6 × 10^5 ^U/L. The freeze–thaw treated cells are a highly active, solid formulation of LmSucP for practical synthesis of GG. An industrial relevant space time yield of 10 g/L/h, according to Woodley et al. [77], was almost achieved (~ 8 g/L/h) even without optimizing synthesis conditions. This underlines the catalyst's applicability for industry. Finally, the current study supports the notion that expression optimization should integrate controlled bioreactor experiments at an early stage of development. The fed-batch cultivations reported here might be even further miniaturized to ~ 1 mL to enhance parallelization capacity [78].

## Materials and methods

### Strain generation

The genes for LmSucP (GenBank identifier: D90314) and BaSucP (GenBank identifier: AF543301) were used in pET21 or pQE30 plasmid vectors. Both genes were codon-optimized for expression in *E. coli* (GenScript Biotech Corp., Piscataway, NJ, USA). *E. coli* Top10F’ was used for plasmid amplification, *E. coli* BL21(DE3) for expression. Cells were transformed by electroporation [79], regenerated in 1 mL SOC medium [80] for 1 h at 37 °C, and selected on lysogeny broth (LB)-agar plates containing 100 mg/L ampicillin.

### Cell preparation

*E. coli* strains were grown at 37 °C in LB-medium (5 g/L NaCl, 5 g/L yeast extract, 10 g/L peptone from casein) in baffled shake flasks containing 100 mg/L ampicillin. The main culture (250 mL in 1 L flasks) was inoculated with cells from an overnight grown preculture (50 mL in 300 mL flasks). Biomass was grown to OD_600_ of 0.8–1.0 and expression was induced with 0.25 mM isopropyl-β-d-thiogalactopyranoside (IPTG). Incubation was overnight at 25 °C and 110 rpm in incubation shaker CERTOMAT BS-1 (Sartorius, Göttingen, DE). OD_600_ was measured spectrophotometrically (DU^®^ 800 UV/Vis Spectrophotometer, Beckman Coulter, Brea, CA, USA). Cell dry mass (CDM) was determined by filtering cell culture (15–20 mL) over pre-weighed Whatman^®^ Nuclepore™ TrackEtched membrane (diameter 50 mm, pore size 0.4 µm, polycarbonate, Sigma Aldrich/Merck, Darmstadt, DE). Washed cells (10–15 mL water) were dried overnight at 70 °C and weighed. Centrifuged cells (20 min, 4 °C, 4.4 krcf, Ultracentrifuge Sorvall RC-5B Superspeed) were resuspended in 50 mM MES buffer, pH 7.0 (6:1 cell wet weight, v:w), aliquoted (~ 15 mL portions) and stored at − 80 °C until further use. Non-permeabilized and freeze-dried cells were processed immediately without an intermediate freezing step.

To prepare the cell lysate, an aliquot of the cell suspension was thawed and ultra-sonicated (Branson Ultrasonics™ Microtips Probe 1/8ʺ dia 418-A, Thermo Fisher Scientific Inc., Waltham, MA, USA) using three times a 6-min run (2 s pulse on, 4 s pulse off, 30% amplitude). Centrifuged cell extract (21.1 krcf, 4 °C for 45 min, Centrifuge Eppendorf 5424R, Eppendorf, Hamburg, DE) was immediately used for enzyme activity determination and subsequently stored at − 20 °C.

### Expression analysis

Enzyme activities were determined in phosphorolysis direction by a continuous coupled activity assay [81]. The αGlc1-*P* liberated on enzyme action was converted by phosphoglucomutase from rabbit muscle (3 U/mL, Sigma-Aldrich/Merck, Darmstadt, DE) and NAD^+^-dependent d-glucose-6-phosphate dehydrogenase from *Leuconostoc mesenteroides* (3.4 U/mL, Sigma-Aldrich/Merck, Darmstadt, DE) to NADH, which then was monitored spectrophotometrically at 340 nm (DU^®^ 800 UV/Vis Spectrophotometer, Beckman Coulter, Brea, CA, USA, see Additional file 1 for details). The protein concentration was determined according to the manual of Carl Roth GmbH (Karlsruhe, DE). The sample (10 μL) was mixed with 500 μL of the fivefold diluted Roti^®^-Qant 5× concentrate (Carl Roth GmbH). After 15 min incubation at room temperature, the absorbance was measured at 595 nm (DU^®^ 800 UV/Vis Spectrophotometer). The protein concentration was calculated from a calibration curve in the range of 0.1 to 1.0 g/L protein (bovine serum albumin, Sigma-Aldrich/Merck, Darmstadt, DE). To obtain comparable activity measurements, the total protein concentration was considered the basis for calculating the activity (Units/mg total soluble *E. coli* protein). The portion of enzyme in total soluble *E. coli* protein was calculated from the specific activities of cell-free extract and isolated enzyme.

Overexpression of the enzymes was additionally checked by SDS PAGE. Ten microlitre of the sample was loaded on a NuPAGE 4–12% Bis–Tris Protein Gel (Thermo Fisher Scientific Inc., Waltham, MA, USA). The protein separation was performed at 175 V for 75 min in 1× MOPS buffer (NuPAGE MOPS SDS Running Buffer 20× , Invitrogen). The finished gel was stained with staining solution (75:500:425; acetic acid:ethanol:water; v:v:v; 2.5 g/L of Brilliant blue R250) for ~ 30 min and destained (75:200:725; acetic acid:ethanol:water, v:v:v) to visualize the protein bands.The portion of insoluble protein relative to soluble protein was determined by band intensity measurements using ImageJ. Lane profile plots were generated by the “gel analysis method” outlined in the ImageJ documentation [82]. See Additional file 1: Figure S3 for one representative SDS PAGE image and corresponding lane profile plots.

### Fed-batch bioreactor cultivations

Cells were grown in a 1.5 L (1.2 L working volume, 0.4 L minimal volume) DASGIP^®^ Parallel Bioreactor System (Eppendorf AG, Hamburg, DE) equipped with a pH probe (Hamilton Bonaduz AG, Bonaduz, CH), an optical DO probe (Hamilton Bonaduz AG), and a DASGIP® GA4X-module (Eppendorf AG) for online off-gas monitoring. The pH was maintained at 7.0 ± 0.05 with 12.5% ammonia solution (Thermo Fisher Scientific, Waltham, USA). The temperature was 37 ± 0.5 °C during the batch phase and decreased to 30 ± 0.5 °C at the beginning of the feed phase. The recombinant production was initiated after 6 h from feed start and the temperature was set to 28 ± 0.5 °C and 25 ± 0.5 °C, respectively. The dissolved oxygen (O_2_) level was stabilized at ≥ 30% saturation by controlling the stirrer speed and the aeration rate. Foaming was suppressed with the antifoam suspension Glanapon 2000 (Bussetti & Co GmbH, Vienna, AT).

The composition of the batch and the fed-batch medium used for cultivation is described elsewhere [83]. All media components were added in relation to the calculated grams cell dry mass (CDM) to be produced and the specific yield coefficient (Y_x/s_ = 0.3 g_biomass_/g_substrate_) for BL21(DE3). Ampicillin (100 mg/L) was added to avoid plasmid loss. Pre-cultures were grown in semi synthetic media and thereof 25 OD_600_ units (25/OD_600_ = volume in mL) were transferred aseptically to the bioreactors. Feeding was initiated when the culture entered the stationary phase (10 g/L CDM in 0.6 L batch medium). A fed-batch regimen with exponential carbon-limited substrate feed was used to provide a constant growth rate of µ = 0.05 h^−1^ throughout 38 h or 2.74 doublings. The substrate feed was controlled by increasing the pump speed according to the exponential growth algorithm, $\mathrm{x}={\mathrm{x}}_{0}{\mathrm{e}}^{\mathrm{\mu t}}$, with superimposed feedback control of weight loss in the substrate bottle. Expression of LmSucP was induced 10 h after feed start, by adding 2 µmol IPTG per g calculated CDM to achieve protein production for two generations.

For off-line analysis (OD_600_, CDM, product), samples were taken before induction, and 24, 26, and 28 h after induction (Fig. 6). To describe cell growth, OD_600_ and CDM were determined according to literature [84]. *E. coli* cell mass was harvested by centrifugation at 18.6 krcf for 15 min and the supernatant was discarded. The cell pellet was stored at − 20 °C for later use.

### Whole cell preparations

To generate non-permeabilized cells, the cell suspension (~ 1 g_*CDM*_/mL 50 mM MES buffer) was stored at 4 °C overnight.

Freeze–thaw treatment: 15 mL of cell suspension (~ 1 g_*CDM*_/mL 50 mM MES buffer) were put to − 80 °C in a 50 mL Falcon tube without any pretreatment. Before usage, cells were thawed at room temperature.

Freeze-dry treatment: The non-permeabilized cell suspension was aliquoted in 1 mL portions to a 24-well plate, flash frozen with liquid nitrogen and freeze-dried at a temperature of − 40 °C and a pressure of ~ 0.2 mbar overnight (Alpha 1–4 LDplus, Martin Christ Gefriertrocknungsanlagen GmbH, Osterode am Harz, DE.

### Glucosylglycerol synthesis in whole cell and cell-free systems

All bioconversions were performed in 100 mL borosilicate glass bottles equipped with Rotilabo^®^ magnetic stirrer bars (25 × 8 mm, Carl Roth GmbH, Karlsruhe, DE). The reaction volume was 50 mL containing 300 mM sucrose, 1800 mM glycerol, 40 U/mL sucrose phosphorylase and 50 mM MES buffer. The reaction was performed at 30 °C (incubation shaker CERTOMAT BS-1, Sartorius, Göttingen, DE) and 300 rpm for 24 h on a Variomag^®^ Multi-Magnetic Stirrer (Thermo Fisher Scientific Inc. Walthman, MA, USA). The reaction was started by adding the cell suspension. The cell catalyst amount was calculated based on the specific activity of the corresponding cell-free extract. The reaction was stopped by heat treatment (99 °C for 5–10 min) (ThermoMixer C, E-5048, Eppendorf, Hamburg, DE). The precipitated protein and/or cell debris was removed by centrifugation for 10 min at 21.1 krcf (Centrifuge Eppendorf 5424 R, Eppendorf, Hamburg, DE) and the supernatant was stored at − 20 °C until HPLC measurements were performed.

### HPLC analysis of reaction compounds

1-GG, GG, sucrose, glycerol, fructose, and glucose in the reaction mixtures were measured by HPLC, either on a Merck Hitachi L-7100 system (Merck, Darmstadt, DE) or a Shimadzu LC-20AD (Kyōto, JPN), both equipped with an autosampler and RI-detector. The stationary phase was a YMC-Pack Polyamine II/S-5 µm/12 nm column (250 mm × 4.6 mm), additionally, a guard column (20 mm × 4.0 mm) was installed (both from YMC Co., Ltd., Shimogōy-ku, Kyōto, JPN). Elution was performed isocratically with an acetonitrile–water mixture (75:25, v:v) at a flow rate of 1 mL/min. Measurements were performed at room temperature, the injection volume per sample was set to 20 µL and the run time was 30 min. Refractive index detection was used. Peaks were analyzed using the software Chromeleon Chromatography Data System (Thermo Fischer Scientific Inc., Waltham, MA, USA). Calibration was done with standards containing 1-GG, GG, sucrose, glycerol and fructose.

## Supplementary Information

**Additional file 1**. Continuous coupled activity assay.** Figure S1.** Plasmids for sucrose phosphorylase expression. (A) pET21: T7_*lacO*_ promoter is regulated by the repressor protein LacI. (B) pQE30: T5_*lacO*_ promoter drives transcription, LacI is lacking. **Figure S2.** Maximum specific growth rates (µ_max_). (A) LmSucP_pQE30, (B) LmSucP_pET21, (C) BaSucP_pQE30, (D) BaSucP_pET21. **Figure S3**. A) Representative SDS PAGE gel for densitometric analysis with the software ImageJ (https://imagej.nih.gov/ij/). The recombinant protein bands of BaSucP (57.50 kDa) and LmSucP (56.81 kDa) are marked with a red arrow. 10 µL of stated dilutions from 0.7 OD units were loaded. B) Lane profile plots. **Figure S4.** SDS PAGE of (A) LmSucP and (B) BaSucP expressed by pQE30 and pET21. The recombinant protein bands of BaSucP (57.50 kDa) and LmSucP (56.81 kDa) are marked with a red arrow. 0.7 OD units were loaded. **Table S1.** Performance metrics comparison of cell-free extracts containing either BaSucP or LmSucP from pQE30. **Figure S5.** SDS PAGE gel of LmSucP shake-flask cultivations at 28 °C expression temperature. 0.7 OD units were loaded. **Figure S6.** SDS PAGE gels of fed-batch bioreactor cultivations at (A) 25 °C and (B) 28 °C expression temperatures.

## Data Availability

The datasets supporting the conclusions of this article are available in the zenodo repository, https://doi.org/10.5281/zenodo.4361383.

## Abbreviations

GG: 2-*O*-α-d-glucosyl-*sn*-glycerol
BaSucP: Sucrose phosphorylase from *Bifidobacterium adolescentis*
LmSucP: Sucrose phosphorylase from *Leuconostoc mesenteroides*
CDM: Cell dry mass
1GG: 1-*O*-α-d-glucosyl-*sn*-glycerol
αGlc1-*P*: α-d-glucose 1-phosphate
