# Supplementary material for: Whole cell-based catalyst for enzymatic production of the osmolyte 2-O-α-glucosylglycerol
Source: Microb Cell Fact. 2021 Apr 7;20:79. doi: 10.1186/s12934-021-01569-4 (PMC8025525; doi:10.1186/s12934-021-01569-4)
Supplement: Supplementary file 1 — Additional file 1. Continuous coupled activity assay. Figure S1. Plasmids for sucrose phosphorylase expression. (A) pET21: T7lacO promoter is regulated by the repressor protein LacI. (B) pQE30: T5lacO promoter drives transcription, LacI is lacking. Figure S2. Maximum specific growth rates (µmax). (A) LmSucP_pQE30, (B) LmSucP_pET21, (C) BaSucP_pQE30, (D) BaSucP_pET21. Figure S3. A) Representative SDS PAGE gel for densitometric analysis with the software ImageJ (https://imagej.nih.gov/ij/). The recombinant protein bands of BaSucP (57.50 kDa) and LmSucP (56.81 kDa) are marked with a red arrow. 10 µL of stated dilutions from 0.7 OD units were loaded. B) Lane profile plots. Figure S4. SDS PAGE of (A) LmSucP and (B) BaSucP expressed by pQE30 and pET21. The recombinant protein bands of BaSucP (57.50 kDa) and LmSucP (56.81 kDa) are marked with a red arrow. 0.7 OD units were loaded. Table S1. Performance metrics comparison of cell-free extracts containing either BaSucP or LmSucP from pQE30. Figure S5. SDS PAGE gel of LmSucP shake-flask cultivations at 28 °C expression temperature. 0.7 OD units were loaded. Figure S6. SDS PAGE gels of fed-batch bioreactor cultivations at (A) 25 °C and (B) 28 °C expression temperatures. [file 12934_2021_1569_MOESM1_ESM.pdf]

Additional file

Whole cell-based catalyst for enzymatic production of the osmolyte 2-*O*-α-glucosylglycerol

Author information

Katharina N. Schwaiger, katharinaschwaiger@acib.at

Monika Cserjan-Puschmann, monika.cserjan@boku.ac.at

Gerald Striedner, gerald.striedner@boku.ac.at

Bernd Nidetzky, bernd.nidetzky@tugraz.at

Abbreviations:

PGM phosphoglucomutase; **G6P-DH** NAD^+-dependent D-glucose-6-phosphate dehydrogenase

Content

Continuous coupled activity assay…..……………………………………………………………………2

Figures S1 and S2.………………………………………………………………………………………3

Figure S3……...…………………………………………………………………………………………..4

Figure S4…………….………..…………………………………………………………………………..5

Table S1 and Figure S5…………………………………………………………………………………6

Figure S6……...…………………………………………………………………………………………..7

References………………………………………………………………………………………………..8

Continuous coupled activity assay

The αGlc1-*P* liberated on enzyme action was converted by phosphoglucomutase (PGM) from rabbit muscle (3.0 U/mL Sigma Aldrich/Merck, Darmstadt, DE) and NAD^+-dependent D-glucose-6-phosphate dehydrogenase (G6P-DH) from *Leuconostoc mesenteroides* (3.4 U/mL, Sigma-Aldrich/Merck, Darmstadt, DE) to NADH, which then was monitored spectrophotometrically at 340 nm over time (DU® 800 UV/Vis Spectrophotometer, Beckman Coulter, Brea, CA, USA).

The assay was performed at 30°C for 15 min and every 16.8 s the absorbance was measured. The absorbance per minute (ΔE/Δt) was obtained and the actual activity (U/mL) was calculated by using the relationship, U/mL =$\Delta E/\Delta t\ V_{total}$ /(V_{sample} ε d). V_{total} is the total volume of the assay mixture (564 µL). The volume of the sample (V_{sample}) was 10 µL, the molar absorption coefficient for NADH (ε) is determined as 6.22 mM^{-1} cm^{-1} and d (1 cm) is the cuvette light path. One unit (U) was defined as the amount of enzyme which produced 1 µM αGlc1-*P* per minute.

The assay mixture consisted of 50 mM potassium phosphate buffer (10 mM $MgCl_2$, 10 mM EDTA, pH 7.0 and 10 µM glucose 1,6-bisphophate, which was added freshly), 3 mM NAD^+ (dissolved in water), 3.4 U/mL G6P-DH dissolved in 3.2 M $(NH_4)_2SO_4$, 50 mM Tris buffer, 1 mM $MgCl_2$, pH 7.0, 3.0 U/mL PGM in 3.2 M $(NH_4)_2SO_4$, 0.001% EDTA pH 6.0 and 10 µL of the properly diluted (in MES buffer) cell-free extract (sample). The reaction was started by adding 250 mM sucrose.

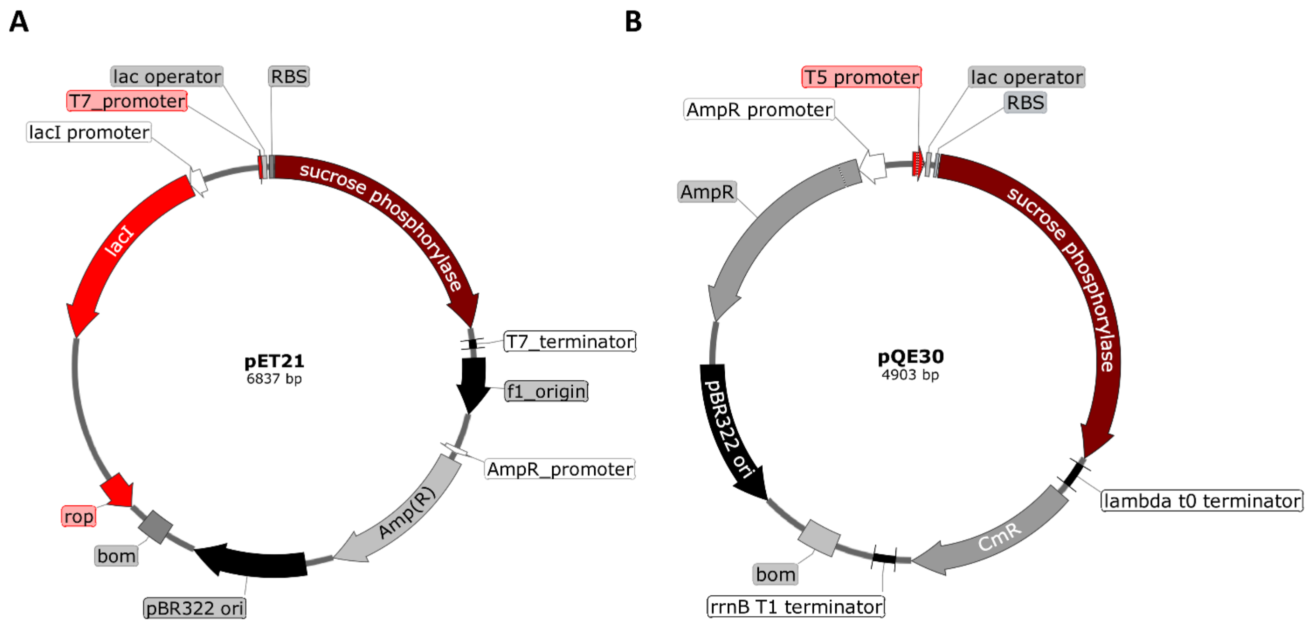

Figure S1: Plasmids for sucrose phosphorylase expression. **(A)** pET21: T7*lacO* promoter is regulated by the repressor protein LacI. **(B)** pQE30: T5*lacO* promoter drives transcription, LacI is lacking.

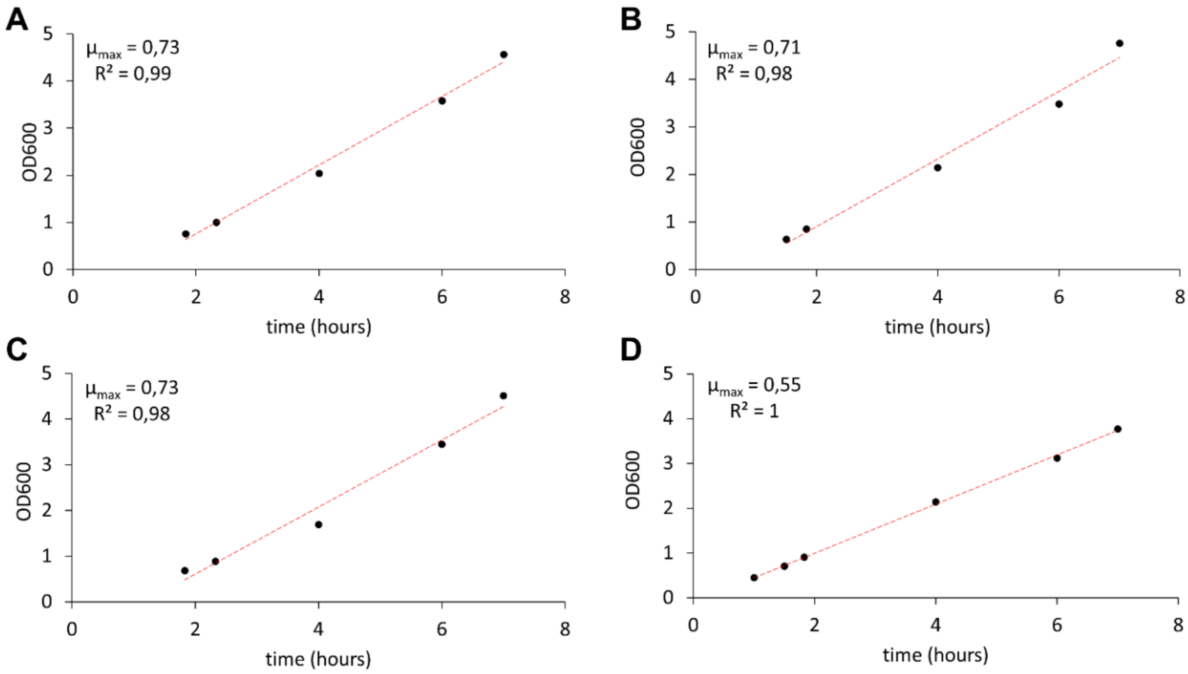

Figure S2: Maximum specific growth rates (μ_{max}). (**A**) LmSucP_pQE30, (**B**) LmSucP_pET21, (**C**) BaSucP_pQE30, (**D**) BaSucP_pET21

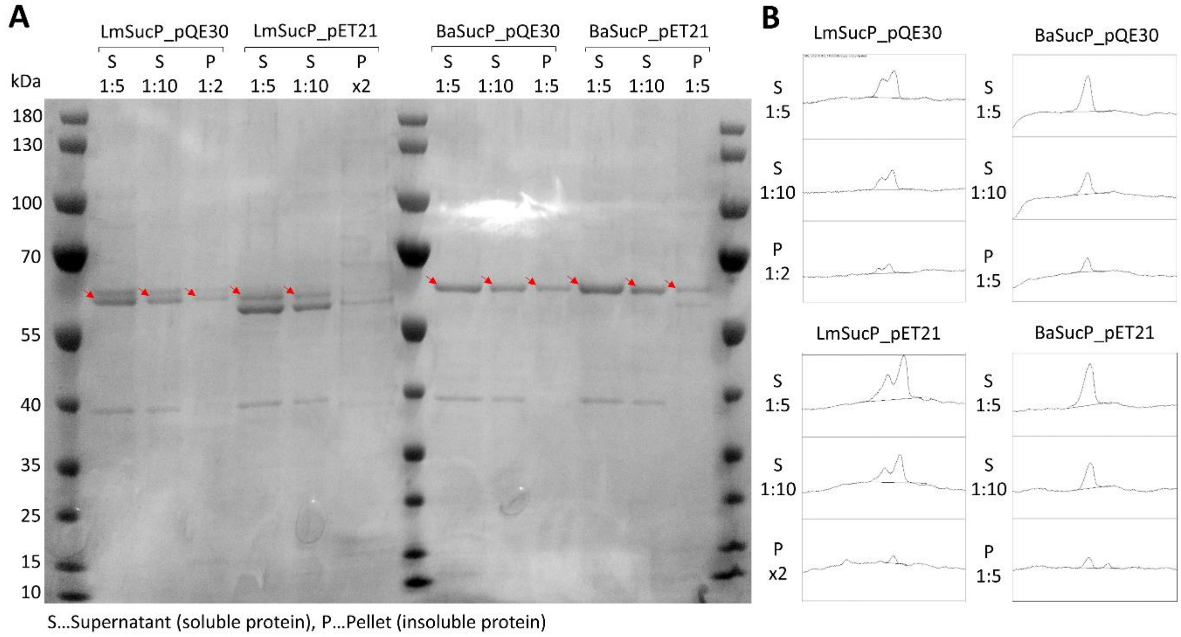

Figure S3: **A**) Representative SDS PAGE gel for densitometric analysis with the software ImageJ (https://imagej.nih.gov/ij/). The recombinant protein bands of BaSucP (57.50 kDa) and LmSucP (56.81 kDa) are marked with a red arrow. 10 µL of stated dilutions from 0.7 OD units were loaded. **B)** Lane profile plots. ***Comment****: The origin of the double bands (LmSucP) was not pursued. However, we assume that an insufficient transcriptional termination* [1] *could be the reason for the larger band (LmSucP_pQE30). The smaller band (LmSucP pET21) might be caused by his-tag cleavage* [2].

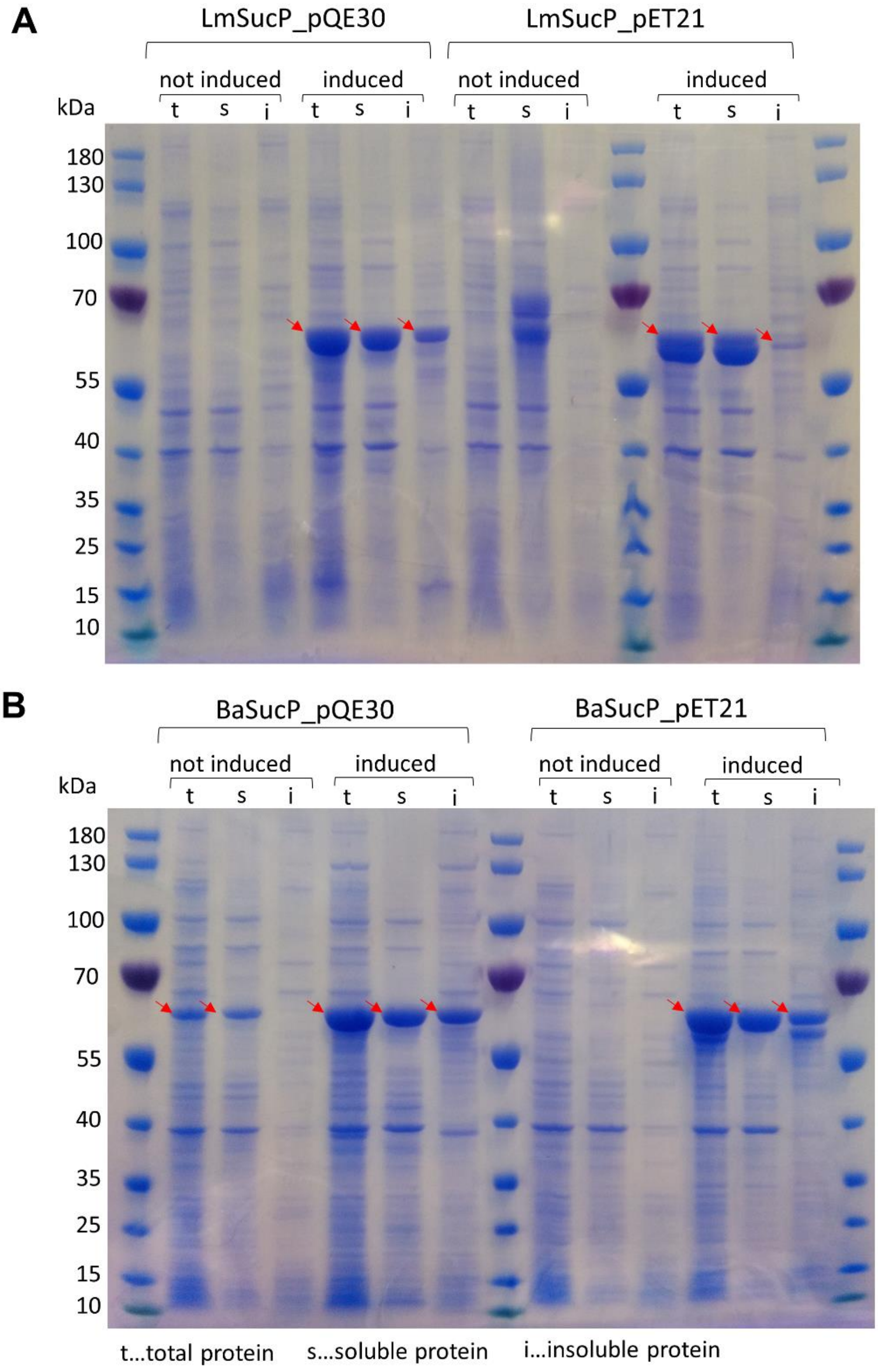

Figure S4: SDS PAGE of **(A)** LmSucP and **(B)** BaSucP expressed by pQE30 and pET21. The recombinant protein bands of BaSucP (57.50 kDa) and LmSucP (56.81 kDa) are marked with a red arrow. 0.7 OD units were loaded.

Table S1: Performance metrics comparison of cell-free extracts containing either BaSucP or LmSucP from pQE30.

	**2-αGG yield**	**cell catalyst**	**product titre**	**STY**	**TTN**
	%	*U/g E. coli protein*	*g/L*	*g/L/h*	*g product g^{-1} E. coli protein*
**LmSucP**	79	1563	68	7.5	97
**BaSucP**	62	485	48	5.6	37

One unit (U) of activity is the enzyme amount producing 1 µmol 2-αGG/min under the conditions used.

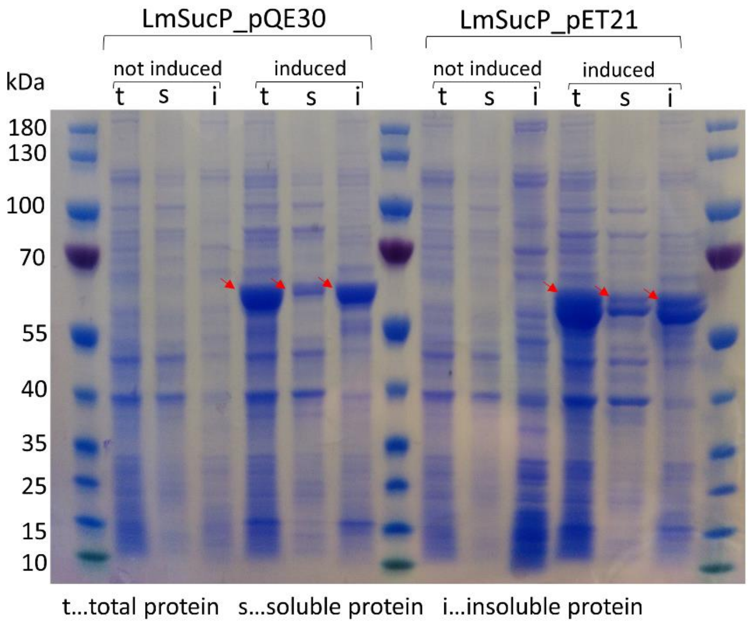

Figure S5: SDS PAGE gel of LmSucP shake-flask cultivations at 28 °C expression temperature. 0.7 OD units were loaded.

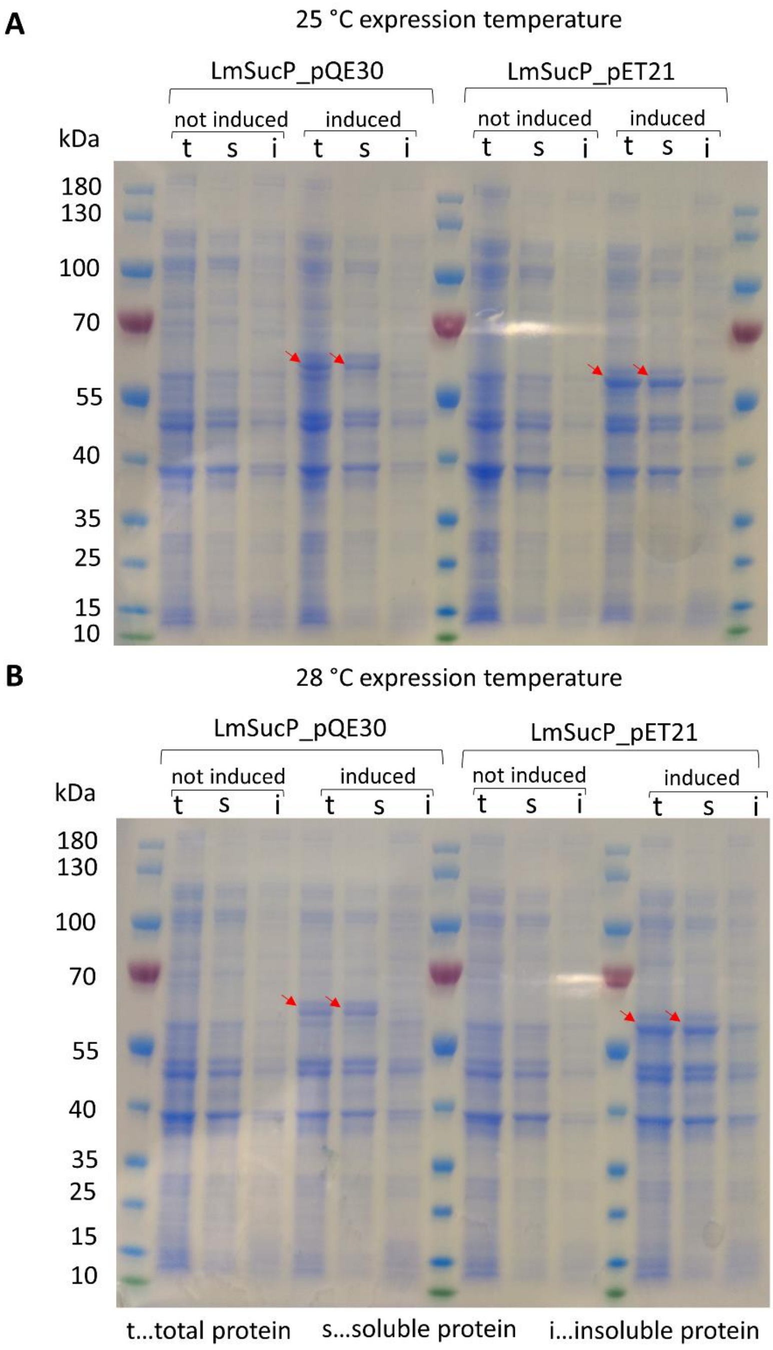

Figure S6: SDS PAGE gels of fed-batch bioreactor cultivations at **(A)** 25 °C and **(B)** 28 °C expression temperatures.

References

[1] Santangelo TJ, Artsimovitch I. Termination and antitermination: RNA polymerase runs a stop sign. Nat Rev Microbiol. 2011;9:319–29.

[2] Lykkemark S, Mandrup OA, Friis NA, Kristensen P. Degradation of C-terminal tag sequences on domain antibodies purified from *E. coli* supernatant. MAbs. 2014;6:1551–9.
